# Influence of Aerobic Training on the Reduced Vasoconstriction to Angiotensin II in Rats Exposed to Intrauterine Growth Restriction: Possible Role of Oxidative Stress and AT_2_ Receptor of Angiotensin II

**DOI:** 10.1371/journal.pone.0113035

**Published:** 2014-11-18

**Authors:** Vanessa Oliveira, Eliana Hiromi Akamine, Maria Helena C. Carvalho, Lisete Compagno Michelini, Zuleica Bruno Fortes, Tatiana Sousa Cunha, Maria do Carmo Franco

**Affiliations:** 1 Nephrology Division, School of Medicine, Federal University of São Paulo, São Paulo, Brazil; 2 Pharmacology Department, University of São Paulo, São Paulo, Brazil; 3 Physiology Department, University of São Paulo, São Paulo, Brazil; 4 Science and Technology Institute, Federal University of São Paulo, São Paulo, Brazil; 5 Physiology Department, School of Medicine, Federal University of São Paulo, São Paulo, Brazil; Max-Delbrück Center for Molecular Medicine (MDC), Germany

## Abstract

Intrauterine growth restriction (IUGR) is associated with impaired vascular function, which contributes to the increased incidence of chronic disease. The aim of this study was to investigate whether aerobic training improves AngII-induced vasoconstriction in IUGR rats. Moreover, we assess the role of superoxide dismutase (SOD) isoforms and NADPH oxidase-derived superoxide anions in this improvement. Female Wistar rats were randomly divided into two groups on day 1 of pregnancy. A control group was fed standard chow *ad libitum*, and a restricted group was fed 50% of the *ad libitum* intake throughout gestation. At 8 weeks of age, male offspring from both groups were randomly assigned to 4 experimental groups: sedentary control (SC), trained control (TC), sedentary restricted (SRT), and trained restricted (TRT). The training protocol was performed on a treadmill and consisted of a continuous 60-min session 5 days/week for 10 weeks. Following aerobic training, concentration–response curves to AngII were obtained in endothelium-intact aortic rings. Protein expression of SOD isoforms, AngII receptors and the NADPH oxidase component p47^phox^ was assessed by Western blot analysis. The dihydroethidium was used to evaluate the *in situ* superoxide levels under basal conditions or in the presence of apocynin, losartan or PD 123,319. Our results indicate that aerobic training can prevent IUGR-associated increases in AngII-dependent vasoconstriction and can restore basal superoxide levels in the aortic rings of TRT rats. Moreover, we observed that aerobic training normalized the increased p47^phox^ protein expression and increased MnSOD and AT_2_ receptor protein expression in thoracic aortas of SRT rats. In summary, aerobic training can result in an upregulation of antioxidant defense by improved of MnSOD expression and attenuation of NADPH oxidase component p47^phox^. These effects are accompanied by increased expression of AT_2_ receptor, which provide positive effects against Ang II–induced superoxide generation, resulting in attenuation of AngII-induced vasoconstriction.

## Introduction

Several approaches that reverse endothelial dysfunction have important applications in a variety of cardiovascular diseases (CVDs). Various pharmacological and nonpharmacological interventions can improve endothelial function [1]–[4]. For example, an epidemiological study suggests that aerobic exercise training is associated with lower morbidity and mortality due to CVDs [5]. Although the mechanisms involved in the reduction of cardiovascular morbidity by aerobic exercise training remain unclear, the reduction of blood pressure levels, modulation of the renin-angiotensin system (RAS), improvement of vascular function, and maintenance of redox balance appear to contribute to decreasing cardiovascular risk [6]–[10].

In recent decades, some metabolic and cardiovascular comorbidities have been associated with an unfavorable intrauterine environment. Several insults (e.g., diet manipulation, hormonal treatments, or surgical procedures) during fetal life can contribute to the development of CVDs in adulthood [11]–[15]. The occurrence of these insults during a critical period of development can result in intrauterine growth restriction (IUGR) and lead to fetal programming [11]–[15]. IUGR significantly correlates with high blood pressure levels, abnormalities in RAS components, and endothelial dysfunction [16]–[18]. In this regard, these comorbidities perpetuate a vicious positive-feedback cycle that can lead to subsequent CVD development.

There is a growing recognition of the need to reduce the negative effects of IUGR. Although clear beneficial roles exist for aerobic exercise in the development and progression of cardiometabolic disease, this influence has been poorly investigated in an experimental model of IUGR. Exercise training has been previously reported to improve insulin sensitivity and restores deficits in pancreatic β-cell mass associated with IUGR in adult rats [19]–[20]. However, the mechanisms underlying the beneficial effects of aerobic exercise training on blood pressure and vascular function in IUGR rats remain unclear.

Thus, we evaluated whether aerobic training improves AngII-induced vasoconstriction in IUGR rats. Moreover, we assess the role of superoxide dismutase (SOD) isoforms and NADPH oxidase-derived superoxide anions in this improvement.

## Materials and Methods

### Animal Care and Protocol

All experimental procedures were approved by the Ethical Committee for Animal Research (Approval Number: 0092/10) at the Federal University of São Paulo and conformed to the guidelines for ethical conduct in the care and use of animals established by the Brazilian Society of Laboratory Animal Science (SBCAL/COBEA). During all of the procedures, the rats were housed in a constant room temperature environment; with a 12∶12 h light–dark cycle and free access to standard rat chow and tap water. The female rats were mated overnight with male breeders (age range of 12–14 weeks) that were obtained from colonies maintained at the Institute of Biomedical Sciences of the University of São Paulo, and the day on which spermatozoa were found in the vaginal smear was designated as the day of conception (day 0). Pregnant rats were transferred to individual standard cages and randomly allocated into one of two groups: the control group (C, n = 10) was fed with a standard chow laboratory animal diet (Nuvilab CR1 - based on the recommendation of the National Research Council and National Institutes of Health, USA) *ad libitum*, and the restricted group (RT, n = 16) was fed with 50% of the typical daily food intake determined by the amount of food consumed by the C group through the gestation period. The amount of food provided daily was weighed, and food intake was monitored. At least four females from the RT group were unable to complete the gestation period due to miscarriages during pregnancy and/or occurrence of fetal resorption, and were excluded from the present study. To prevent any variation in neonatal growth due to the availability of milk intake during suckling, the litter size was standardized to eight pups, and the gender ratio was kept as close to 1∶1 as possible. The offspring was nursed by their mothers until weaning at day 21, after that was fed with a standard chow laboratory animal diet. Beginning at 8 weeks of age, male offspring from each experimental group (C = 40; RT = 40) were randomly assigned to 4 experimental groups: sedentary control (SC, n = 20 from 10 litters), trained control (TC, n = 20 from 10 litters), sedentary restricted (SRT, n = 20 from 12 litters), and trained restricted (TRT, n = 20 from 12 litters).

### Aerobic Training Protocol

Rats were initially adapted to walking/running on a motor treadmill (8 to 10 sessions at 0.4 up to 0.6 km/h; 0% grade; 10 minutes). Subsequently, rats were subjected to a maximal exercise test (graded exercise with increments of 0.3 km/h every 3 minutes, beginning at 0.3 km/h up to the maximal intensity attained for each animal), which was necessary to establish the intensity of the aerobic training. The aerobic training protocol was performed over 10 weeks, 5 day/week, with intensity between 50–60% (low-intensity training) of the maximal capacity test. The duration was progressively increased until 1 hour/day. At 5 weeks, the maximal capacity test was repeated to readjust the running speed. The untrained animals were handled every day and subjected once per week to a period of aerobic exercise (0.4 up to 0.6 km/h; 0% grade; 10 minutes). This approach has been used in order of submitting untrained animals to the same handled stress that the trained rats, however, it is unable to promote the aerobic training adaptations.

### Measurement of Blood Pressure (BP) and Heart Rate (HR)

Resting BP and HR were noninvasively determined using a computerized tail-cuff system (PowerLab 4/S ADInstruments Pty Ltd, Castle Hill, Australia) prior to and following the aerobic training protocol. Rats were acclimatized to the apparatus during daily sessions over 5 days, 1 week prior to the commencement of the experimental period.

### Vascular Reactivity Study

Forty-eight hours after the last training session, the rats were decapitated and vascular function was evaluated. Segments of the thoracic aorta (4 mm in length), which were free of connective tissue, were mounted in an isolated tissue chamber containing Krebs–Henseleit solution, which consisted of 118 mM NaCl, 4.7 mM KCl, 25 mM NaHCO_3_, 2.5 mM CaCl_2_·2H_2_O, 1.2 mM KH_2_PO_4_, 1.2 mM MgSO_4_·7H_2_O, 11 mM glucose, and 0.01 mM EDTA. The solution was gassed with 95% O_2_–5% CO_2_ and was maintained at a resting tension of 1.5 g at 37°C and pH 7.4. Isometric tension was recorded using an isometric force transducer (Letica TRI 210, Barcelona, Spain) connected to an acquisition system (PowerLab 8/30, ADInstruments Pty Ltd, Castle Hill, Australia). After 60 min of equilibration, concentration–response curves to angiotensin II (AngII, 10^−9^ to 3×10^−5 ^M) were obtained in endothelium-intact aortic rings. Vascular responses to AngII are expressed as grams of tension. At the end of the experiment, the presence of functional endothelium was verified in all aortic rings by observing whether relaxation occurred upon exposure to acetylcholine (ACh, 10^−5 ^M). The endothelium was considered intact if the aortic ring relaxed more than 80% upon ACh treatment. The maximal response (R_max_) and the log agonist concentration resulting in 50% of the R_max_ (log EC_50_) were calculated for each concentration-response curve using nonlinear regression analysis (GraphPad Prism Software, USA).

### Detection of Superoxide Anions in the Aorta

The oxidation-sensitive fluorescent dye dihydroethidium (DHE) was used to evaluate the *in situ* concentration of superoxide, as previously described [21]. Thoracic aortas were carefully dissected and cleaned of connective tissue. Aortas were then divided into cylindrical segments of 4 mm in length and were first immersed in an embedding medium (tissue freezing medium) and then frozen and stored at −80°C until the determination of superoxide anion levels. Transverse aortic sections (7 µm) were obtained in a cryostat from the previously frozen aortas, collected on glass slides, and allowed to reach equilibrium for 30 min at 37°C in phosphate-buffered saline (PBS). Fresh PBS containing DHE (5 µmol L^−1^) was topically applied to each tissue section and incubated in a light-protected, humidified chamber at 37°C for 30 min. Control sections received an identical volume of PBS. Some assays were performed in the presence of AngII (10^−4 ^M), apocynin (an inhibitor of NADPH oxidase, 10^−4 ^M), losartan (an AT_1_ receptor antagonist, 10^−4 ^M), or PD 123,319 (an AT_2_ receptor antagonist, 10^−4 ^M). Images were obtained using a Nikon E1000 microscope equipped for epifluorescence (excitation at 488 nm; emission at 610 nm). Fluorescence was detected using a 585-nm long-pass filter. The fluorescence intensity was quantified using ImageJ software (Wayne Rasband, National Institutes of Health, USA) and is expressed in arbitrary units. The fluorescence ratio was evaluated in at least three locations in each image.

### Tissue Preparation for Western Blot Analysis

Forty-eight hours after the last training session, the rats were decapitated, and the thoracic aorta samples were harvested, weighed, frozen, stored at −80°C, and used for Western blot analysis. For the analysis of CuZnSOD, MnSOD, and p47^phox^, the total tissue lysates were prepared using lysis buffer [100 mM Tris-HCl at pH 7.4, 100 mM sodium pyrophosphate, 10 mM sodium orthovanadate, 100 mM NaF, 10 mM EDTA, 2 mM phenylmethylsulfonyl fluoride (PMSF), 0.01 mg/mL aprotinin, and 1% Triton X-100]. For the analysis of the AT_1_ and AT_2_ receptors, the aortas were homogenized in a buffer containing 50 mM Tris-HCl at pH 7.4, 1 mM EDTA, 10% sucrose, 2 mM PMSF, 0.01 mg/mL leupeptin, and 0.01 mg/mL aprotinin. Homogenates were centrifuged (15,000 *g*, 30 min, 4°C), and the supernatant was collected. The protein content of the lysates was determined using the BCA (bicinchoninic acid) Protein Assay Kit (Pierce Biotechnology, Rockford, IL, USA). Proteins from homogenized aortas (70 µg of protein extracts) were treated with Laemmli buffer containing 200 mmol/L dithiothreitol (DTT) and then subjected to SDS-PAGE in 10% polyacrylamide gels. Following electrophoresis, proteins were electrotransferred onto PVDF membranes (BioRad, Hercules, USA).

### Western Blot Analysis

The protein expression of CuZnSOD, MnSOD, p47^phox^, and the AT_1_ and AT_2_ receptors was analyzed using Western blot analysis. After blocking nonspecific sites with T-TBS buffer containing 5% nonfat dry milk and 3% bovine serum albumin (BSA) at room temperature for 1 hour, the membranes were incubated overnight at 4°C with the following primary antibodies: anti-CuZnSOD (1∶1000, Upstate Biotechnology, USA); anti-MnSOD (1∶2000, Upstate Biotechnology, USA); anti-p47^phox^ (1∶1500, Upstate Biotechnology, USA); anti-AT_1_ (1∶1000, Proteimax Inc., Brazil); and anti-AT_2_ (1∶1000, Proteimax Inc., Brazil). The primary antibody was detected using peroxidase-conjugated secondary anti-rabbit IgG antibodies (MnSOD and p47^phox^, 1∶4000; CuZnSOD, 1∶2000; AT_1_ and AT_2_, 1∶15,000). Identical membranes were used to determine α-actin protein expression using a mouse monoclonal antibody (1∶1500, Sigma-Aldrich). The immune complexes were detected using an enhanced luminol chemiluminescence system (ECL Plus, GE Healthcare, UK), which was subsequently exposed to a photographic film. The film was developed, and the bands were analyzed using ImageJ software (Wayne Rasband, National Institutes of Health, USA). The total expression of the analyzed proteins was normalized to α-actin protein expression levels and is expressed as the ratio between the optical density of the specific protein and the optical density of α-actin.

### Statistical Analysis

Statistical analysis was carried out using the SPSS (v17, SPSS Inc., Chicago, IL, USA). Concentration–response curves were constructed using Prism (v.6; GraphPad Software, San Diego, CA, USA) and sigmoidal curves fitted to the data using the least squares method. The results are expressed as the means ± S.E.M. The normality of distribution of each variable was tested and transformed data were used when necessary. Where appropriate, data were analyzed using a Student’s unpaired *t* test, two-way ANOVA to determine IUGR/aerobic training interaction or three-way ANOVA to determine IUGR/aerobic training/treatment interaction effects for dependent variables. A subsequent Tukey’s *post-hoc* test was used to examine data for specific intergroup differences. A level of 5% probability was considered significant.

## Results

Maternal nutrient restriction during pregnancy resulted in IUGR, as indicated by a marked reduction in the birth weight (C: 6.8±0.2; RT: 4.1±0.1, n = 40, P<0.001). Higher BP was observed in SRT rats, however, after 10 weeks of aerobic training the BP levels was reduced in these rats (Table 1). Two-way ANOVA revealed significant interaction between IUGR and aerobic training on BP (F (1,34) = 9.491, P = 0.001), indicating that these factors acted dependently. However, the HR significantly decreased after 10 weeks of aerobic training for both TRT and TC rats in comparison to the sedentary groups (Table 1). Two-way ANOVA indicated no significant interaction between IUGR and aerobic training on HR (*P* = 0.736), but very significant main effects for trained (F (1,26) = 23.99, P<0.01).

**Table 1:** Birth Weight, Hemodynamic Measurements, and Maximal Response to Angiotensin II in the experimental groups.

	SC (n)	TC (n)	SRT (n)	TRT (n)	*P* (Two-Way ANOVA)		
					IUGREffect	TrainedEffect	InteractionIUGR×Trained
**Blood Pressure (mmHg)**	111±2 (20)	109±3 (20)	122±3 (20)#	110±2 (20)			0.001
**Heart Rate (bpm)**	321±4 (20)	299±3 (20)‡	325±8 (20)	300±5 (20)^‡‡^		<0.001	
**R_Max_ Ang II (grams of tension)**	0.37±0.66 (7)	0.40±0.03 (7)	0.66±0.03 (7)#	0.38±0.02 (7)	0.002	0.005	0.001

Data are mean ±SEM. (n) number of rats.

# *P*<0.05 *vs.* SC; TC and TRT after a Tukey’s *post hoc* test.

‡ *P*<0.05 *vs. S*C and ^‡‡^ *vs.* SRT after a *Tukey post hoc* test.

**Figure 1:**
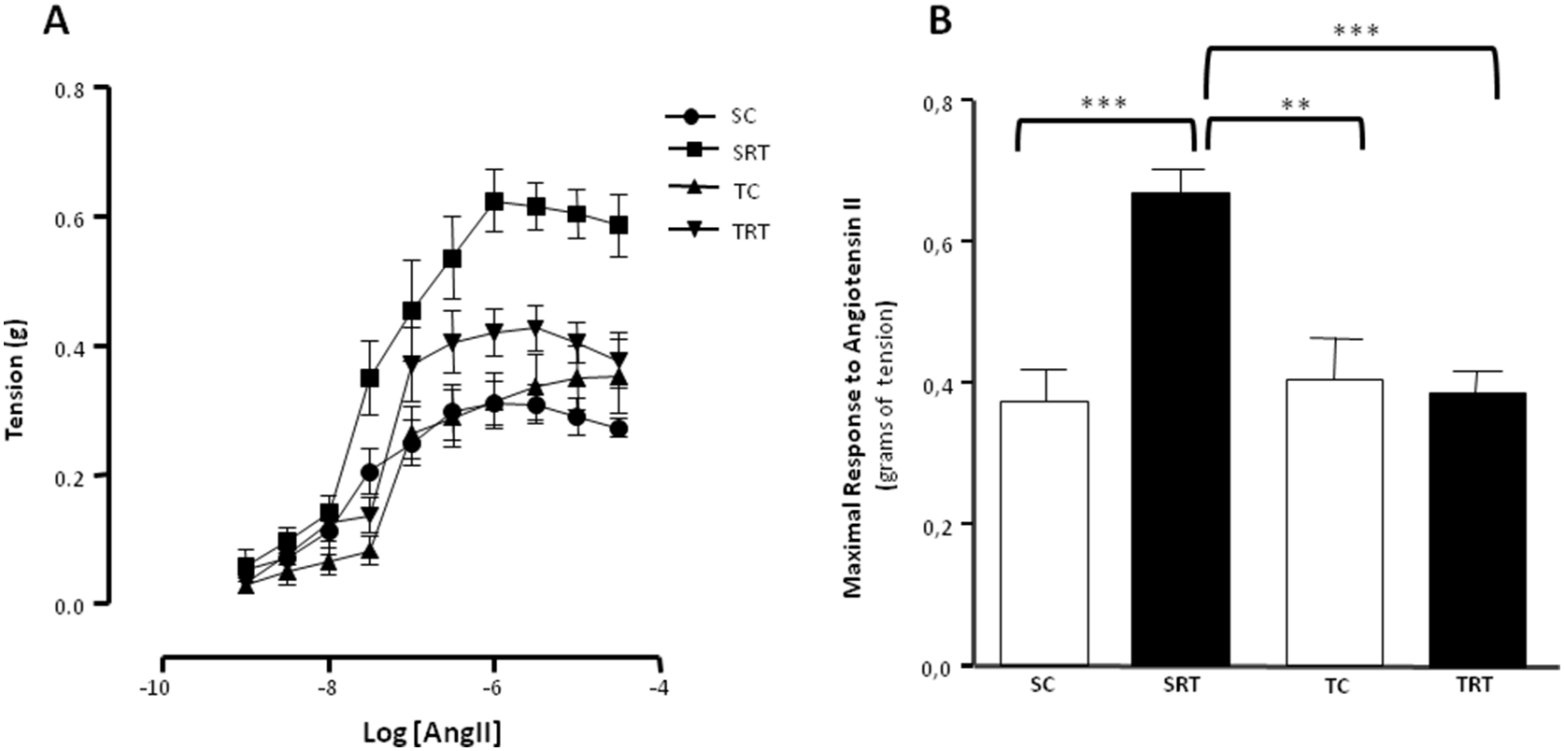
Effect of the IUGR and aerobic training on the vascular reactivity to Angiotensin II. (**A**) Dose-dependent contractions (**B**) Maximal response to angiotensin II in aortic rings isolated from the sedentary control (SC), sedentary restricted (SRT), trained control (TC), and trained restricted (TRT) groups. Values are expressed as the mean ± SEM from seven animals per group. Two-way ANOVA analysis was performed to assess the effects of IUGR, aerobic training and of their interaction (IUGR X Trained) on the aortic contractile responses. **P = 0.001 and ***P<0.001 in Tukey’s post Hoc test.

In comparison to the aortic rings from SC rats, the aortic rings from SRT rats were more responsive to AngII. Aerobic exercise training decreased the hyperreactivity to AngII of aortic rings isolated from TRT rats to levels that are similar to those observed in SC rats, but aerobic exercise training did not alter the corresponding responses in aortic rings from TC rats (Table 1, Figure 1). A significant interactive effect of IUGR and aerobic training on maximal contractile responses to AngII was confirmed by two-way ANOVA (F (1, 25) = 14.4, *P* = 0.001). Moreover, main effects for IUGR (*P* = 0.002) and aerobic training (*P* = 0.005) were also significant.

**Figure 2:**
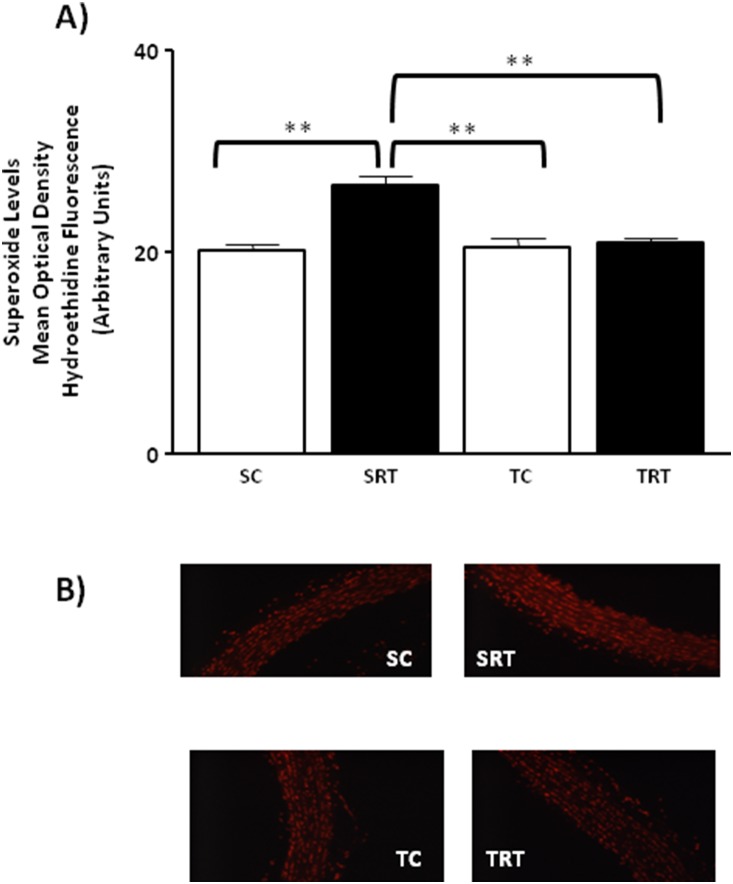
Effect of the IUGR and aerobic training on the vascular superoxide concentration. (**A**) Histogram and (**B**) digital images illustrate the presence of superoxide in dihydroethidium (DHE)-treated sections of aortic rings from SC, SRT, TC, and TRT rats. Values are expressed as the means ± SEM from six animals per group. Two-way ANOVA analysis was performed to assess the effects of IUGR, aerobic training and of their interaction (IUGR X Trained). **P = 0.001 in Tukey’s post Hoc test.

As shown in Figure 2, in DHE fluorescence intensity the two-ANOVA revealed significant IUGR-dependent differences (F (1, 14) = 23.584, *P*<0.001, main effect of IUGR) with highest values found in SRT rats than in SC rats, suggesting increased superoxide production in IUGR rats. Aerobic exercise training for 10 weeks significantly reduced superoxide levels in the aortas of TRT rats. Superoxide production remained unaltered in the aortas of TC rats (Figure 2). Moreover, we found significant interaction between IUGR and aerobic training in superoxide production (F (1, 14) = 17.554, *P* = 0.001).

**Figure 3:**
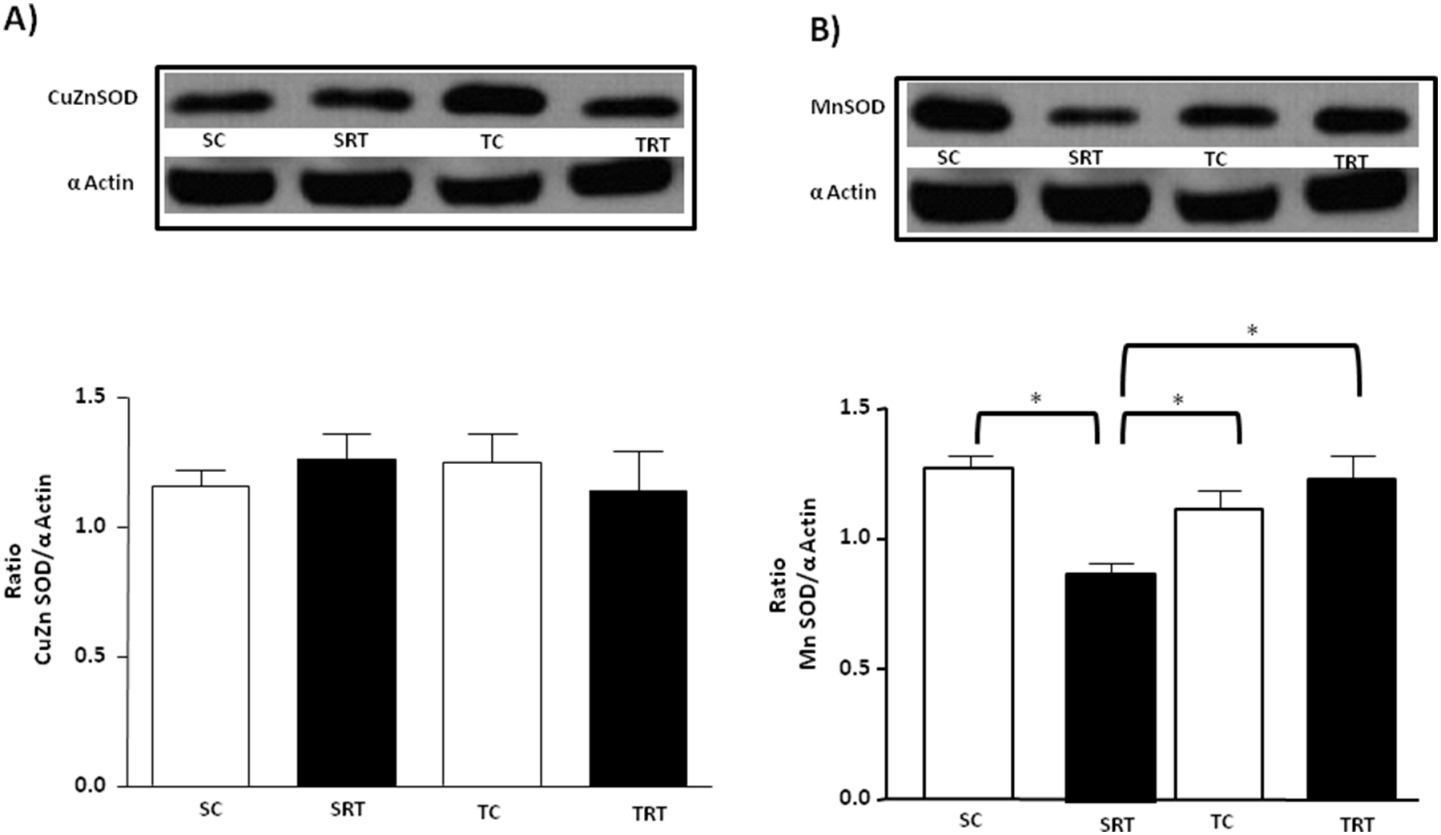
Expression profile of aortic SOD isoforms. Western blot analyses of CuZnSOD (**A**) and MnSOD (**B**) protein expression in thoracic aortas from SC, SRT, TC, and TRT rats. Representative immunoblots and the corresponding optical density normalized to the optical density of α-actin. Values are expressed as the mean ± SEM from five animals per group. Two-way ANOVA analysis was performed to assess the effects of IUGR, aerobic training and of their interaction (IUGR X Trained). *P<0.05 in Tukey’s post Hoc test.

**Figure 4:**
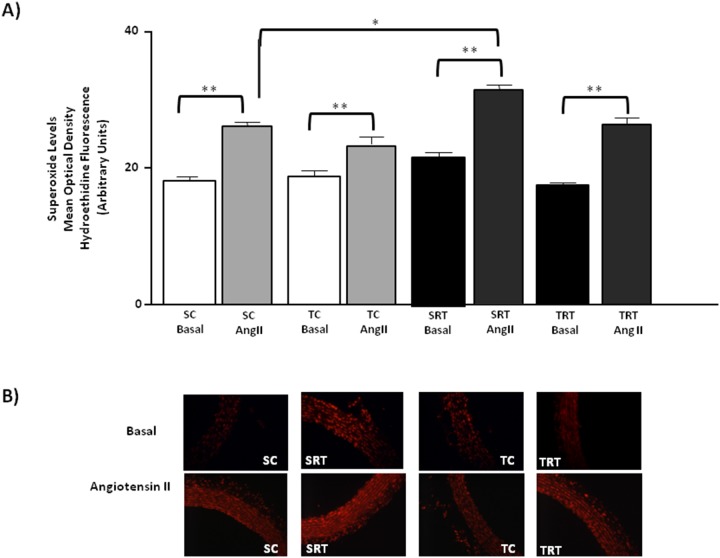
Role of the angiotensin II stimulation on the vascular superoxide production. (**A**) Histogram and (**B**) digital images illustrate the presence of superoxide in dihydroethidium (DHE)-treated sections of aortic rings from SC, SRT, TC, and TRT rats under basal conditions and following stimulation with angiotensin II (Ang II: 10^−4 ^M). Three-way ANOVA analysis was performed to assess the effects of IUGR, aerobic training, Ang II treatment and of their interactions. Values are expressed as the mean ± SEM from six animals per group. **P = 0.001 and *P<0.05 in Tukey’s post Hoc test.

**Figure 5:**
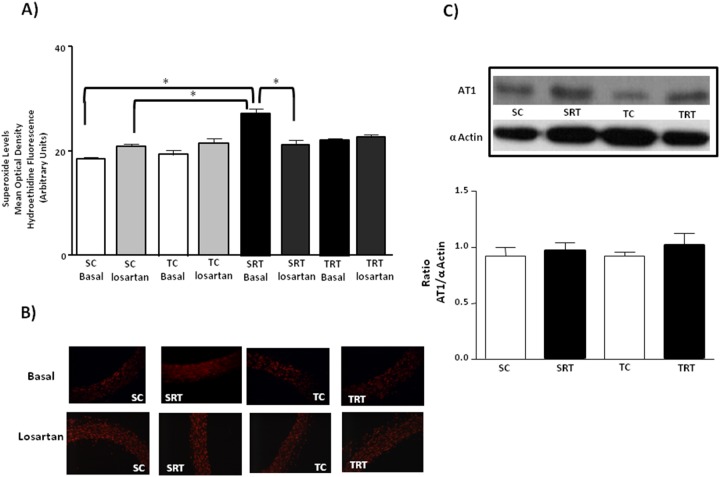
Effect of the AT_1_ receptor antagonist on the vascular superoxide production and expression profile of aortic AT_1_ receptor. (**A**) Histogram and (**B**) digital images illustrate the presence of superoxide in dihydroethidium (DHE)-treated sections of aortic rings from SC, SRT, TC, and TRT rats under basal conditions and in the presence of losartan (Los: 10^−4 ^M). Three-way ANOVA analysis was performed to assess the effects of IUGR, aerobic training, losartan treatment and of their interactions. (**C**) Representative immunoblots and the corresponding optical density of AT_1_ receptors normalized to the optical density of α-actin. Two-way ANOVA analysis was performed to assess the effects of IUGR, aerobic training and of their interaction. Values are expressed as the mean ± SEM from six animals per group. *P<0.05 in Tukey’s post Hoc test.

As shown in Figure 3, we did not observe any significant differences in the protein expression of CuZnSOD among all groups. However, MnSOD protein expression was significantly decreased in SRT rats compared with SC rats, whereas this alteration was normalized by aerobic exercise training (Figure 3B). In contrast, MnSOD protein expression was significantly decreased in SRT rats compared with SC rats, whereas this alteration was normalized by aerobic exercise training (Figure 3B). A significant interaction between IUGR and aerobic training was observed for the MnSOD protein expression (F (1, 16) = 4.290, *P* = 0.05) and main effects for IUGR (*P* = 0.022) and aerobic training status (*P* = 0.034). In all experimental groups, we observed that treatment of the aorta segments with AngII promoted an increase in superoxide levels, although this effect was greater in aortic rings isolated from SRT rats (Figure 4). This was confirmed by three-way ANOVA that indicated significant main effect of IUGR (F (1, 29) = 27.350, *P*<0.001) and treatment (F (1, 29) = 28.671, *P*<0.001) on superoxide production. As shown in Figure 5, treatment of the aorta segments with losartan significantly reduced basal superoxide levels in the aortas of SRT rats. Three-way ANOVA revealed significant IUGR-dependent differences in superoxide concentration (F (1,30) = 9.797, *P* = 0.004, main effect of IUGR) with IUGR/treatment interactions (F (1,30) = 15.087, *P* = 0.001). Furthermore, IUGR or aerobic exercise training did not alter the protein expression of the AT_1_ receptor in the isolated thoracic aortas (Figure 5C).

**Figure 6:**
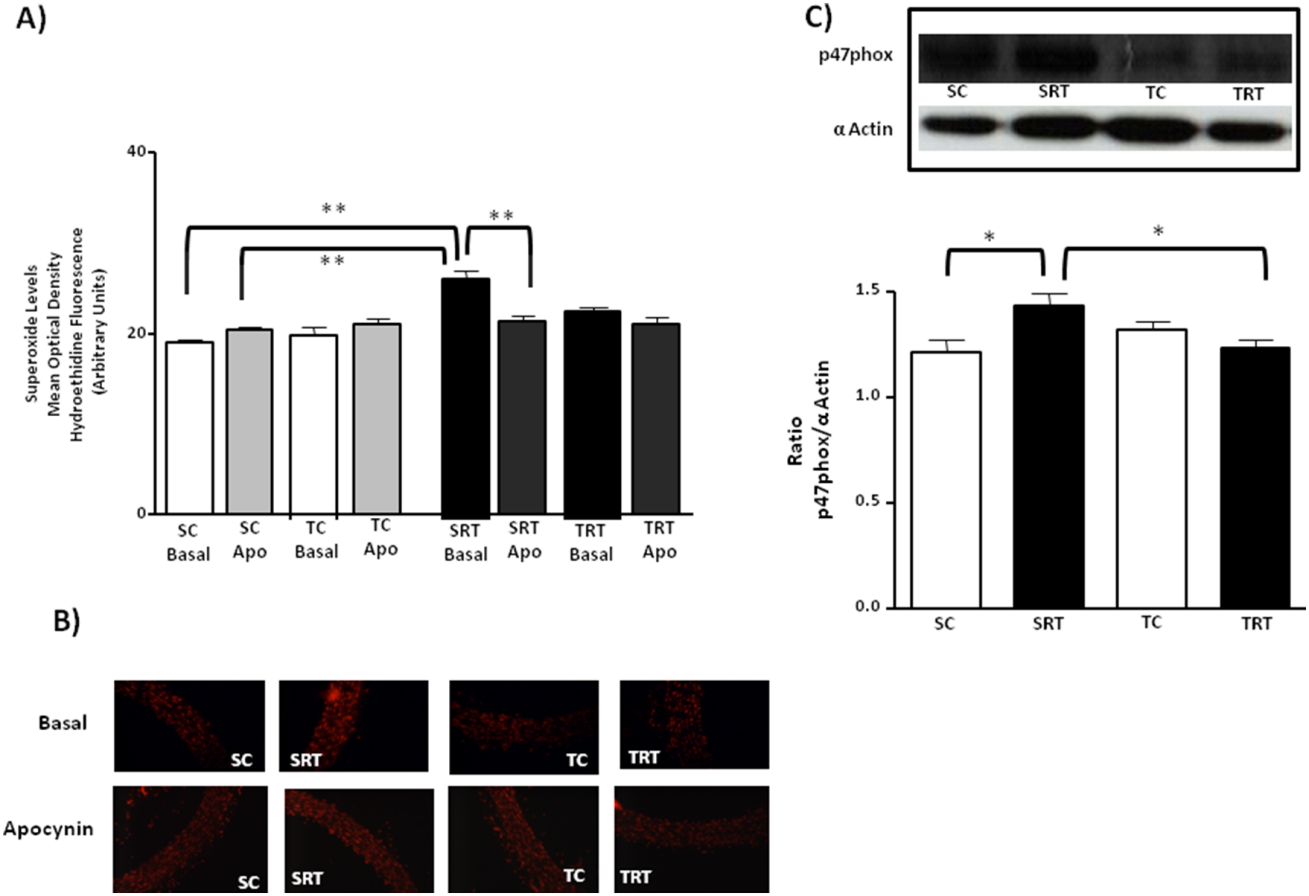
Effect of the NADPH-oxidase inhibitor on the vascular superoxide production and protein expression of aortic NADPH subunit. (**A**) Histogram and (**B**) digital images illustrate the presence of superoxide in dihydroethidium (DHE)-treated sections of aortic rings from SC, SRT, TC, and TRT rats under basal conditions and in the presence of apocynin (Apo: 10^−4 ^M). Three-way ANOVA analysis was performed to assess the effects of IUGR, aerobic training, apocynin treatment and of their interactions. (**C**) Representative immunoblots and the corresponding optical density of the p47^phox^ subunit normalized to the optical density of α-actin. Two-way ANOVA analysis was performed to assess the effects of IUGR, aerobic training, apocynin treatment and of their interaction. Values are expressed as the mean ± SEM from six animals per group. **P = 0.001 and *P<0.05 in Tukey’s post Hoc test.

To determine whether NADPH oxidase was involved in the production of superoxide, we evaluated the effect of the inhibitor apocynin. Indeed, treatment with apocynin reduced basal superoxide production in only SRT rats, suggesting that vascular NADPH oxidases may participate in this production of superoxide (Figure 6). This was confirmed by three-way ANOVA between groups analysis that showed a significant main effect for IUGR (F (1,30) = 25.809, *P*<0.001) with IUGR/treatment interaction (F (1,30) = 19.280, *P*<0.001). Apocynin inhibits the release of superoxide anions by NADPH oxidase by preventing the translocation of the p47^phox^ subunit to the membrane. The aortas from SRT rats exhibited increased p47^phox^ protein expression (Figure 6C). However, aerobic exercise training reduced p47^phox^ protein expression in the aortas of TRT rats but did not alter p47^phox^ protein expression in TC rats (Figure 6). In this case two-way ANOVA revealed a significant interaction between IUGR and aerobic training on p47^phox^ protein expression (F (1,16) = 7.807, *P* = 0.013).

**Figure 7:**
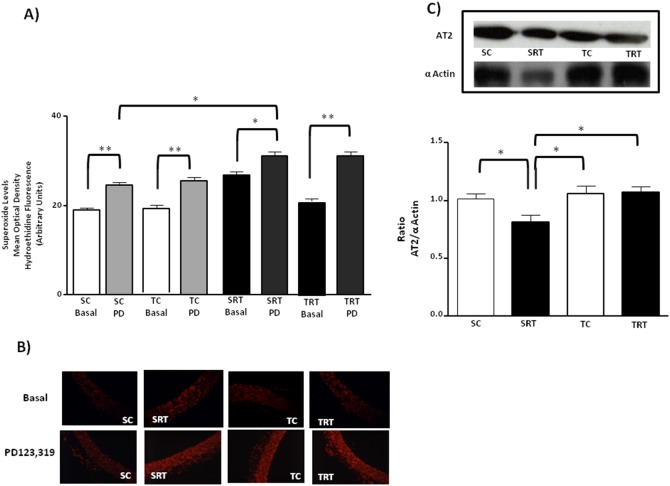
Effect of the AT_2_ receptor antagonist on the vascular superoxide production and expression profile of aortic AT_2_ receptor. (**A**) Histogram and (**B**) digital images illustrate the presence of superoxide in dihydroethidium (DHE)-treated sections of aortic rings from SC, SRT, TC, and TRT rats under basal conditions and in the presence of PD 123,319 (PD: 10^−4 ^M). Three-way ANOVA analysis was performed to assess the effects of IUGR, aerobic training, PD 123,319 treatment and of their interactions. (**C**) Representative immunoblots and the corresponding optical density of AT_2_ receptors normalized to the optical density of α-actin. Two-way ANOVA analysis was performed to assess the effects of IUGR, aerobic training and of their interaction. Values are expressed as the mean ± SEM from six animals per group. **P = 0.001 and *P<0.05 in Tukey’s post Hoc test.

**Figure 8:**
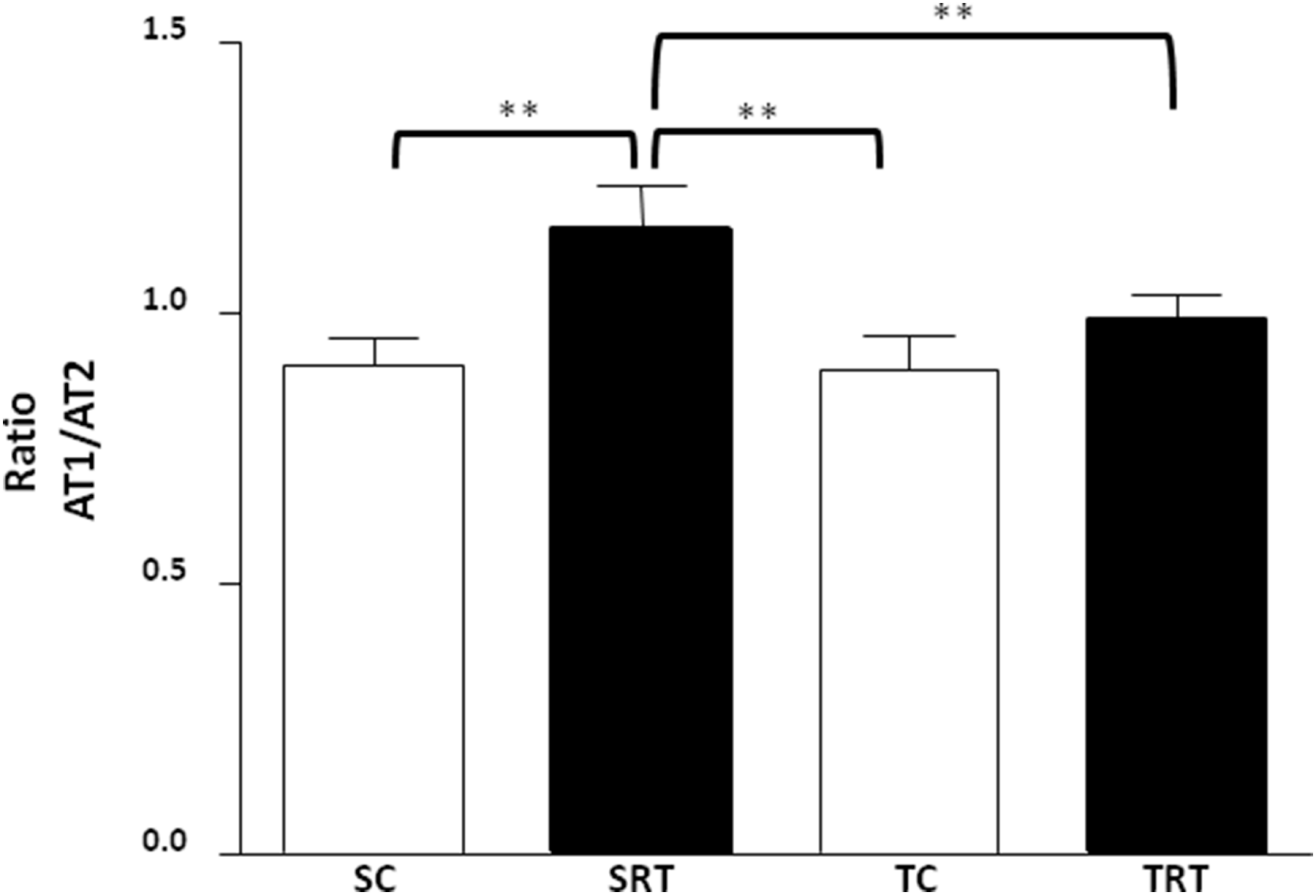
Representative histogram of the AT_1_/AT_2_ ratio in thoracic aortas of SC, SRT, TC, and TRT rats. Values are expressed as the mean ± SEM from six animals per group. Two-way ANOVA analysis was performed to assess the effects of IUGR, aerobic training and of their interaction (IUGR X Trained). **P = 0.001 in Tukey’s post Hoc test.

Given that AT_2_ receptors exert a beneficial vascular effect, we further examined superoxide levels following antagonism with the AT_2_ receptor antagonist PD 123,319. Interestingly, we observed increased superoxide levels following PD 123,319 treatment in only SRT and TRT rats, whereas PD 123,319 treatment did not demonstrate an effect in SC or TC rats (Figure 7). Three-way ANOVA did not reveal a significant interaction between IUGR/aerobic training/treatment on superoxide concentration. Instead, main effects were confirmed for IUGR (F (1,32) = 23.095, *P*<0.001) and PD 123,319 treatment (F (1,32) = 14.004, *P* = 0.001), indicating that these factors acted independently. To gain additional insight into this response, we investigated the protein expression of AT_2_ receptors. We observed that AT_2_ protein levels were reduced in the aortas of SRT rats and were restored following aerobic exercise training, whereas AT_2_ protein expression in TC rats was unaltered (Figure 7C). A significant interaction between IUGR and aerobic training affected the AT2 protein expression in aorta (F (1,15) = 4.540, *P* = 0.050). Additionally, the AT_1_/AT_2_ ratio was significantly increased in SRT rats compared with SC rats (Figure 8), whereas aerobic exercise training resulted in the normalization of AT_1_/AT_2_ levels in TRT rats. No changes were observed in the AT_1_/AT_2_ ratio in TC rats. Consistent with these results, the two-way ANOVA also reveal significant interaction between IUGR and aerobic exercise training on ratio (F (1,15) = 4.955, *P* = 0.042).

## Discussion

It is well recognized that IUGR can lead to several pathologies including hypertension, type 2 diabetes mellitus, and cardiovascular disorders [11]–[18]. In contrast, aerobic exercise training promotes several beneficial cardiovascular adaptations [6]–[10]. This study highlights five important findings. First, aerobic exercise training can prevent the IUGR-associated increase in AngII-dependent vasoconstriction in the aortic rings of TRT rats. Second, exercise training reversed the increase in basal levels of superoxide in TRT rats. Third, exercise training reversed the downregulation of MnSOD protein expression in TRT rats. Fourth, aerobic exercise training significantly upregulated AT_2_ receptor protein expression in TRT rats. Fifth, exercise training significantly attenuated p47^phox^ protein expression, which is a subunit of NADPH oxidase.

In this study, we found that the thoracic aorta vasoconstriction to AngII were higher in SRT rats. The modulation of AngII-induced vasoconstriction exhibited a biphasic response, in which the most potent vasoconstriction occurs at lower concentrations of AngII. Additionally, we found that aerobic exercise training for 10 weeks attenuates AngII-induced vasoconstriction in aortic rings from TRT rats. Although the possible beneficial effect of aerobic exercise training on the endothelial dysfunction induced by IUGR was not investigated in this study, the notion that exercise training primarily affects the modulatory function of endothelial cells is supported by findings in animal models of hypertension [22], diabetes [23], aging [24], and acute myocardial infarction [25]. However, the mechanisms involved in this beneficial effect on IUGR-induced vascular dysfunction remain unclear.

Increasing studies suggest that superoxide anions are not only implicated but also play an important role in the endothelial dysfunction induced by IUGR [26]–[28]. Scavengers of superoxide anions and treatment with other antioxidants have been shown to improve IUGR-related endothelial dysfunction by restoring NO bioavailability [26], [28]. In this study, aerobic exercise training for 10 weeks significantly reduced superoxide generation, but only in TRT rats. Several studies have demonstrated that an antioxidant effect is induced by exercise training *via* the enhancement of the activity of radical scavenger enzymes [29]–[33]. Aerobic training decreases oxidative stress by increasing the efficiency of the antioxidant system in isolated arteries from SHR [32] and diabetic [33] rats. Consistent with these findings, our results indicated that MnSOD protein expression levels were decreased in SRT rats in comparison to SC rats, and aerobic exercise reversed this downregulation. Numerous studies indicate that MnSOD is abundantly expressed in endothelial cells compared with other cell types, suggesting that MnSOD protects against endothelial dysfunction [34]–[37]. Our results support the hypothesis that the positive effect of aerobic exercise observed in the TRT rats is, in part, due to an enhanced capacity of MnSOD to scavenge superoxide.

The ability of AngII to stimulate the vascular production of superoxide by mediating NADPH oxidase activation has been widely recognized [38]–[40]. Our results support this finding by a marked increase in superoxide production following AngII treatment, which is inhibited by the addition of both losartan and apocynin in SRT rats. Additionally, we observed that AT_1_ receptor protein expression was unaltered by IUGR or by aerobic exercise training. Conversely, we found that the protein expression of the NADPH oxidase subunit p47^phox^ was higher in SRT rats, whereas aerobic exercise training was effective in normalizing p47^phox^ expression. Physical training performed prior to lung ischemia/reperfusion has been reported to be effective in normalizing p47^phox^ protein expression in mesenteric arteries [41]. p47^phox^ plays an important role in superoxide production in response to AngII [42]. Recently, it has been shown the effects of IUGR on AngII-mediated activation of the NADPH oxidase complex [29]. Results presented here suggest that Ang II-mediated generation of superoxide in a p47phox-dependent manner in SRT rats, whereas 10 weeks of aerobic exercise training was effective in downregulation of p47phox subunit protein expression.

The activation of AT_1_ receptors is known to promote vasoconstriction through increased smooth muscle intracellular Ca^2+^ availability, whereas AngII binding to AT_2_ receptors on the endothelium causes vasodilation through the activation of the NO-cGMP axis [43], [44]. Our results indicate that an increased response to AngII stimulation occurred with a decrease in AT_2_ receptor protein expression in isolated aortas of SRT rats. Moreover, a higher ratio of AT_1_/AT_2_ receptors was also found in these rats. The role of AT_2_ receptor stimulation in cardiovascular cells is not entirely understood. The AT_2_ receptor is a counter-regulatory component of the RAS, and AngII stimulation of this receptor opposes the effects mediated by the AT_1_ receptor on cell growth, vascular function, and fluid balance [44]. Studies have reported that AT_2_ activation increased NO production, which significantly, but not entirely, contributes to the beneficial effects observed following AT_1_ receptor antagonism [45], [46]. Furthermore, some metabolites of AngII degradation (e.g., Ang (1–7) and Ang III) are known to exert their biological actions, at least partially, by binding to the AT_2_ receptor [47]. This versatility of the AT_2_ receptor appears to be highly cell-type and tissue specific in determining the complex cross-talk between angiotensin receptors and certain metabolites.

A correlation exists between the AT_2_ receptor and aerobic exercise. Some reports indicate an upregulation of AT_2_ receptor protein levels in heart, ventricular tissue, and vessels in several animal models [48]. In this study, we found that aerobic exercise training for 10 weeks restores AT_2_ receptor protein expression in aortic rings from TRT rats. The AT_1_/AT_2_ ratio in the aorta was also normalized by exercise training, thereby locally modifying the actions of AngII. Moreover, we observed that AT_2_ antagonism with PD 123,319 increased superoxide formation, but only in SRT and TRT rats, supporting the inhibitory role of AT_2_ receptors on superoxide production in restricted rats. Knowledge of how AT_2_ mediates superoxide anion generation is limited, and little is known regarding the likely beneficial effects of aerobic exercise on the modulation of this receptor. Studies suggest that AngII receptors differentially modulate endothelial superoxide generation [49]. Sohn *et al*. [49] reported that AT_1_ activates superoxide formation and that AT_2_ receptors appear to attenuate this AT_1_-induced effect, most likely *via* a pathway that involves tyrosine phosphatases. A study by Sabuhi *et al*. [50] suggests that the activation of AT_2_ receptors lowers oxidative stress by increasing SOD activity and decreasing the expression of the NADPH oxidase component gp91^phox^. Our study suggests the possibility of an association between AT_2_ modulation and exercise training. However, the exact mechanisms of these effects remain to be elucidated.

In summary, the results of this study provide evidence that aerobic training demonstrates a beneficial effect on antioxidant capacity *via* the upregulation of MnSOD protein expression. The vascular protective effects of exercise training may partially be due to the downregulation of p47^phox^ and, subsequently, the reduced activation of NADPH oxidase. Finally, the increase in AT_2_ receptor protein levels may also provide beneficial effects against superoxide generation. Nevertheless, we did not directly evaluate the role of nitric oxide (NO) regarding the beneficial effects of exercise training; assessment of this pathway would add considerable clarity to the mechanisms that regulate vascular function and NO–redox–based signaling in IUGR rats submitted to aerobic training.
